# Mechanistic insights into the orthogonal functionality of an AHL-mediated quorum-sensing circuit in *Yersinia pseudotuberculosis*

**DOI:** 10.1016/j.synbio.2024.10.002

**Published:** 2024-10-14

**Authors:** Boyu Luo, Shanshan Wu, Wei Liu, Dongdong Zhang, Ruicun Liu, Tuoyu Liu, Zhi Sun, Ziqun Wei, Mingyu Liu, Zhiyuan Shi, Niu Huang, Yue Teng

**Affiliations:** a State Key Laboratory of Pathogen and Biosecurity, Academy of Military Medical Sciences, Beijing, 100071, China; b National Institute of Biological Sciences, No. 7 Science Park Road, Zhongguancun Life Science Park, Beijing, 102206, China; c Laboratory Department in Second Medical Center of PLA General Hospital, Beijing, 100089, China; d Western Medical Branch of PLA General Hospital, Beijing, 100041, China; e Center for Quantitative Biology, Peking-Tsinghua Center for Life Sciences, Academy for Advanced Interdisciplinary Studies, Peking University, Beijing, 100871, China; f School of Pharmaceutical Science and Technology, Faculty of Medicine, Tianjin University, Tianjin, 300072, China; g Tsinghua Institute of Multidisciplinary Biomedical Research, Tsinghua University, Beijing, 102206, China

**Keywords:** *Yersinia pseudotuberculosis*, Quorum sensing, Molecular dynamics, Genetic circuit, Biosensors

## Abstract

YpsR, a pivotal regulatory protein in the quorum-sensing (QS) of *Yersinia pseudotuberculosis*(*Y. pstb*), is essential for molecular signaling, yet its molecular mechanisms remain poorly understood. Herein, this study systematically investigates the interactions between YpsR and acyl-homoserine lactones (AHLs), shedding light on the selective mechanism of YpsR to various AHL molecules. Using molecular docking and surface plasmon resonance (SPR) analysis, we confirmed YpsR's binding affinities, with the strongest observed for 3OC6-HSL, which notably inhibited *Y. pstb* growth. Additionally, we engineered a whole-cell biosensor based on YpsR-AHL interaction, which exhibited sensitivity to the signal molecule 3OC6-HSL produced by *Y. pstb*. Furthermore, key YpsR residues (S32, Y50, W54, D67) involved in AHL binding were identified and validated. Overall, this research elucidates the mechanisms of QS signal recognition in *Y. pstb*, providing valuable insights that support the development of diagnostic tools for detecting *Y. pstb* infections.

## 1 Introduction

*Yersinia* is a genus of gram-negative bacteria within the *Enterobacteriaceae* family, including three known species pathogenic to humans: *Yersinia pestis*, *Yersinia enterocolitica* and *Yersinia pseudotuberculosis* itself. Among these, *Yersinia pseudotuberculosis* (*Y. pseudotuberculosis, Y. pstb*) is a human intestinal pathogenic bacterium equipped with a flagellar system and motility function [[1], [2], [3]]. This bacterium possesses a broad host range and is primarily transmitted through contaminated water or food, leading to gastrointestinal diseases in mammals and rodents. As a significant zoonotic pathogen shared between humans and animals, *Y. pstb* causes symptoms such as fever, headache, abdominal pain, and vomiting, with severe cases potentially resulting in enteritis and mesenteric lymphadenitis. *Y. pstb* poses a considerable threat to human health and has been implicated in outbreaks in Finland and Japan [[4], [5], [6]].

Bacteria regulate the expression of virulence-, invasion-, and symbiosis-related genes through quorum-sensing (QS) systems [7,8]. These systems are vital for bacterial interactions with hosts, enhancing survival capabilities, and influencing pathogenicity [[9], [10], [11], [12]]. Understanding the cell signaling selection and transduction mechanisms of *Yersinia* QS systems is of paramount importance. Different bacteria utilize various small molecule signaling molecules for quorum sensing, which are known as autoinducers (AI). In Gram-negative bacteria, quorum sensing systems are typically induced by N-acyl-l-homoserine lactones (AHL) signaling molecules. Many Gram-negative bacteria employ LuxI/LuxR-type quorum sensing systems, generating a range of AHL signals, with each organism producing distinct AHL molecules [13]. The first AHL molecule, N-3-oxohexanoyl-l-homoserine lactone (3OC6-HSL), was discovered in marine bacteria *Vibrio fischeri* in the 1980s and was shown to control bacterial bioluminescence [14]. Taking *Vibrio fischeri* as an example, the specific role of AHL in the QS system is as follows: the AHL-induced quorum sensing system comprises three genetic components: the autoinducer synthesis enzyme gene luxI, the transcriptional activator gene luxR, and a bidirectional lux promoter. AHL molecules synthesized by LuxI can freely diffuse across the cell membrane, with their concentration increasing as bacterial density rises. Upon reaching a threshold, AHL binds to LuxR, forming a complex that activates the bidirectional promoter, leading to the transcription of the luciferase genes *luxCDABE* [15]. Previous studies have identified QS systems in the *Yersinia* genus, including Y*ersinia enterocolitica*, *Yersinia pseudotuberculosis*, and *Yersinia pestis*. Both Yersinia *pseudotuberculosis* and Yersinia p*estis* harbor two sets of LuxR/LuxI-type QS systems [16]. Specifically, *Yersinia pestis* expresses *ypeI*/*ypeR* and *yspI*/*yspR*, while *Yersinia pseudotuberculosis* expresses *ypsI*/*ypsR* and *ytbI*/*ytbR*. YpeI and YspI synthases are responsible for synthesizing at least 24 types of acylated homoserine lactones (AHLs). The YspI synthase specifically synthesizes hexanoyl-l-homoserine lactone (C6-HSL), octanoyl-l-homoserine lactone (C8-HSL), N-3-oxo-hexanoyl homoserine lactone (3-oxo-C6-HSL), N-3-oxo-octanoyl homoserine lactone (3-oxo-C8-HSL), and other related compounds [[17], [18], [19]]. Atkinson et al. analyzed the impact of YpsR/I and YtbR/I mutations on the motility of *Y. pstb,* confirming the synergistic action of the YpsR/I and YtbR/I systems [20,21]. They demonstrated that the YpsR/I system negatively regulates itself while positively regulating the YtbR/I system. Additionally, Zhang et al. [22] revealed that YpsI and YtbI synthases positively regulate the type VI secretion system, with added AHLs significantly influencing the expression of type VI secretion system genes. QS also plays a crucial role in bacterial biofilm formation, symbiosis between endophytic bacteria and the host, bacterial–bacterial interactions, and environment adaptability. Following an investigation of the quorum sensing systems of *Yersinia pestis* and relevant literature [[23], [24], [25], [26]] such as LuxR/LuxI, we have identified 10 AHL molecules out of over 24 candidates that may potentially trigger a response in the YpsR/YpsI system. These AHLs include 3OC6, 3OC8, 3OC10, 3OC12, 3OC14, as well as C6, C8, C10, C12, and C14.

Despite these findings, the structural basis for its binding to AHL molecules and its molecular mechanism in regulating downstream proteins require further investigated. This study aims to analyze the YpsR protein, focusing on its molecular signal recognition and binding mechanisms to enhance our understanding of *Y. pstb* pathogenicity-related functions. By leveraging AlphaFold and molecular dynamics simulations, we elucidate the monomeric structure of YpsR and its interactions with homoserine lactone molecules, identified key amino acid residues involved in substrate specificity, determined the binding capacities of the different molecules with YpsR, and assessed their impacts on bacterial growth. Furthermore, we explored the molecular signal recognition mechanisms of YpsR through gene circuitry analysis. By employing YpsR/I and downstream genes, we constructed sensitive and specific whole-cell biosensors capable of detecting pathogens such as *Y. pstb* [[27], [28], [29], [30]]. This research contributes to clarifying the signaling principles of the *Y. pstb* QS system, exploring the mechanisms of cellular functional variations, and providing a theoretical foundation and technical support for the prevention and treatment of *Yersinia* infections.

## 2 Results

### 2.1 Structural insights into the mechanisms of YpsR-AHL interaction

**Fig. 1:**
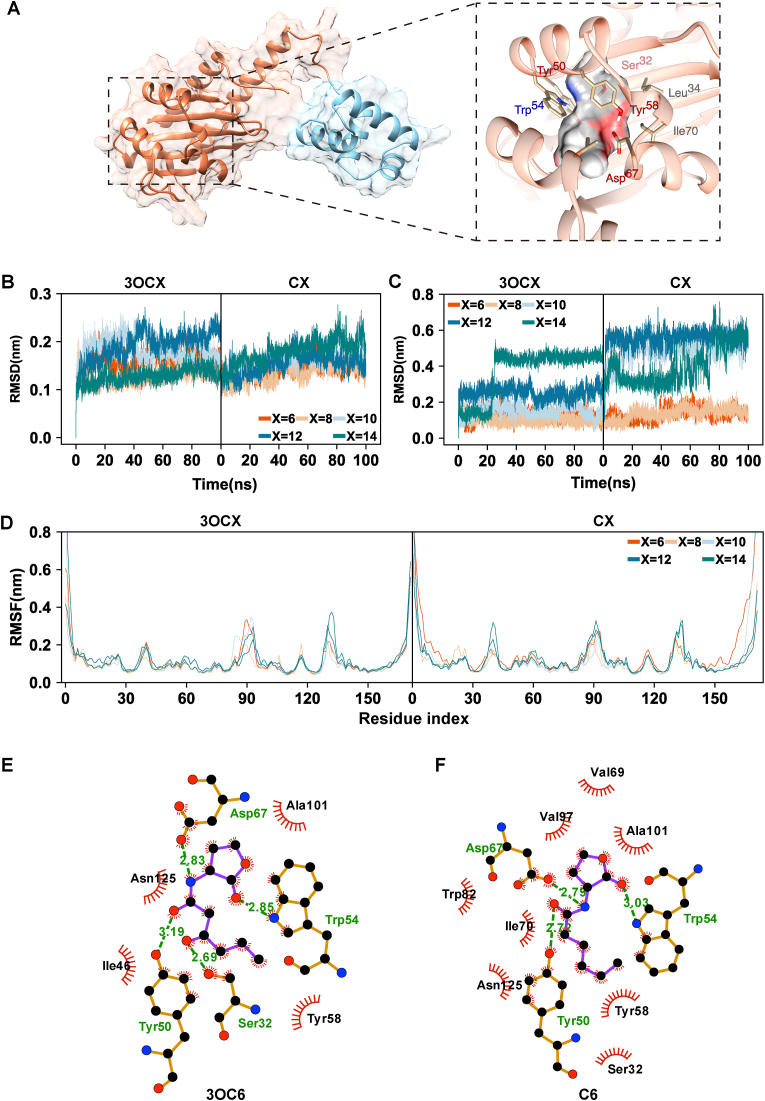
**Overview of the YpsR protein based on AlphaFold prediction.** (A) Depiction of the full-length YpsR protein, with the following color-coded domains: the cytoplasmic membrane-associated regulatory domain (residues 1–179) in coral and the DNA binding domain (residues 180–245) in light blue. (B) Root mean squared deviation (RMSD) as a function of time for the cytoplasmic membrane-associated regulatory domain when YpsR is complexed with different AHLs. (C) RMSD of the different AHLs using the initial binding pose as the reference. (D) Root mean square fluctuation (RMSF) profiles from the MD simulations of the structures of the YpsR cytoplasmic membrane-associated regulatory domain complex with the five different 3OCx AHLs and Cx AHLs. (E–F) Molecular interactions of AHLs with the YpsR protein, (E) 3OC6, (F) C6.

Inspired by our prior works [16], LuxR has demonstrated strong binding affinity with 3OC6-HSL, and we have prior experience using MD simulations to model the binding conformation of *Vibrio fischeri* LuxR with 3OC6-HSL. Therefore, we selected 3OC6-HSL for the MD simulations of the YpsR protein in this study as well. We employed AlphaFold to predict the three-dimensional structure of YpsR, AutoDock Vina to predict the ligand binding pose, and conducted molecular dynamics (MD) to confirm the stability of the final YpsR-3OC6 complex. The cytoplasmic membrane-associated regulatory domain of YpsR features a tertiary structure composed of five β-sheets and four flexible α-helices. The binding site is predominantly hydrophobic, with an apolar solvent-accessible surface area (SASA) of approximately 77 Å^2^. Notably, key residues within the binding site—Trp54, Tyr50, Tyr58, Ser32, and Asp67—appear to participate in crucial hydrogen bond interactions and ligand stabilization (Fig. 1A).

All-atom MD simulations were conducted to investigate the stability of the YpsR regulatory domain cytoplasmic in complex with ten different AHLs (Fig. S1＆Fig. S2A). The stability of the protein and protein–ligand interactions was assessed over 100 ns of MD simulation. The root mean square deviation (RMSD) of the protein backbone (Fig. 1B) exhibited steady behavior, achieving equilibrium shortly after 10 ns. In the presence of C14, the protein RMSD stabilized after 50 ns. Ligand RMSD analysis revealed that 3OCx (x = 6, 8, 10, 12) and Cx (x = 6, 8) exhibited minimal deviation from their initial binding poses, stabilizing quickly during the simulation (Fig. 1C). Conversely, 3OC14 and Cx (x = 10, 12, 14) showed noticeable deviations, suggesting less stable binding interactions or suboptimal conformational predictions. The root mean square fluctuations (RMSFs) obtained from the MD simulations (Fig. 1D) provided further insights into protein conformational dynamics, revealing that the secondary structures forming the binding site remained relatively rigid, with RMSF values of approximately 1 Å. In contrast, the loops connecting the α-helices (residues 85–95 and 128–132) exhibited increased flexibility, accommodating ligands of varying sizes.

Binding poses of AHLs were predicted using AutoDock Vina and refined through subsequent MD simulations. Protein‒ligand interactions were analyzed using the LigPlot + program, which revealed conserved hydrogen bonds between the carbonyl oxygen of AHLs and Trp54 and Tyr50, and between the amide nitrogen and Asp67. Additionally, hydrophobic interactions were facilitated by residues Ile46, Val97, and Ala101. Compared to 3OC6, the absence of the carbonyl group in the C6 ligand led to the loss of a hydrogen bond with Ser32 (Fig. 1E & F), resulting in decreased binding affinity. As the size of AHLs increased, longer chains induced deformation of the binding site, affecting hydrogen-bond interactions. For instance, in the predicted complex conformation of 3OC12, the hydrogen bond with Ser32 was lost (Fig. S2B).

### 2.2 Binding kinetics of YpeR to diverse AHL using surface plasmon resonance

**Fig. 2:**
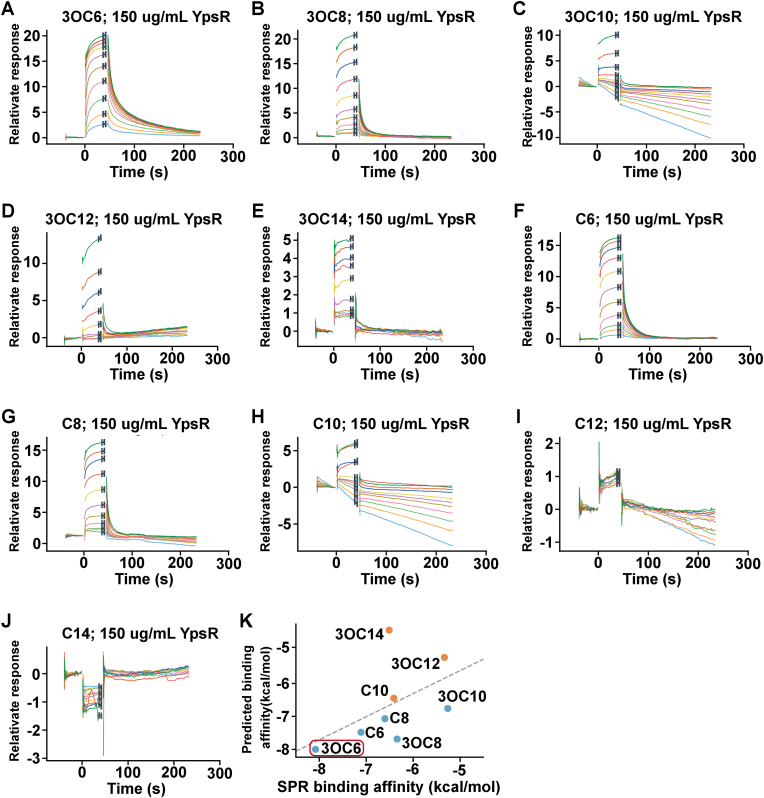
**In vitro experiments verified the affinity of YpsR for 10 AHLs.** (A–J) Surface plasmon resonance characterization of the binding of 10 AHL small molecules to the YpsR 1–179 residue maltose-binding protein; (K) correlation between the experimental and predicted binding affinities calculated using the Vina program.

We next purified YpeR (1–179) from *Escherichia coli* and produced MBP-fused YpsR through a four-step purification method (Fig. S3). Surface plasmon resonance (SPR) was empolyed to determine the equilibrium dissociation constants of ten small-molecule AHLs binding to MBP-fused YpsR, thereby validating our previous predictions. The equilibrium dissociation constants correlated negatively with the affinities of the AHL molecules. A scatter plot in Fig. 2 illustrates the relationships between SPR-measured binding affinities and those predicted by AutoDock Vina docking, revealing a positive correlation with a Pearson correlation coefficient of 0.51. Excluding the outlier prediction 3OC14 increased the correlation coefficient increases to 0.76, underscoring the robustness of the predictive model for protein‒ligand interactions when the ligand size is appropriate for the binding pocket. Table 1 lists the equilibrium dissociation constants for the 10 AHLs, and 3OC6 having the lowest dissociation constant, indicating its strongest affinity for the YpsR regulatory protein. This finding aligns with previous in vivo experiments demonstrating the optimal antibacterial effects of 3OC6.

**Table 1:** The experimental and predicted binding energies.

AHLs	Dissociation constant (μM)	SPR binding energy (kcal/mol)	Predicted binding energy (kcal/mol)
3OC6	1.30	−8.08	−8
C6	6.60	−7.11	−7.5
3OC8	24.20	−6.34	−7.7
C8	15.60	−6.6	−7.1
3OC10	146.00	−5.26	−6.8
C10	21.30	−6.41	−6.5
3OC12	130.00	−5.33	−5.3
C12	nan	nan	−5.8
3OC14	18.20	−6.51	−4.5
C14	nan	nan	−4.8

### 2.3 3OC6-HSL inhibited the growth of *Y. pstb* by interacting with YpsR

**Fig. 3:**
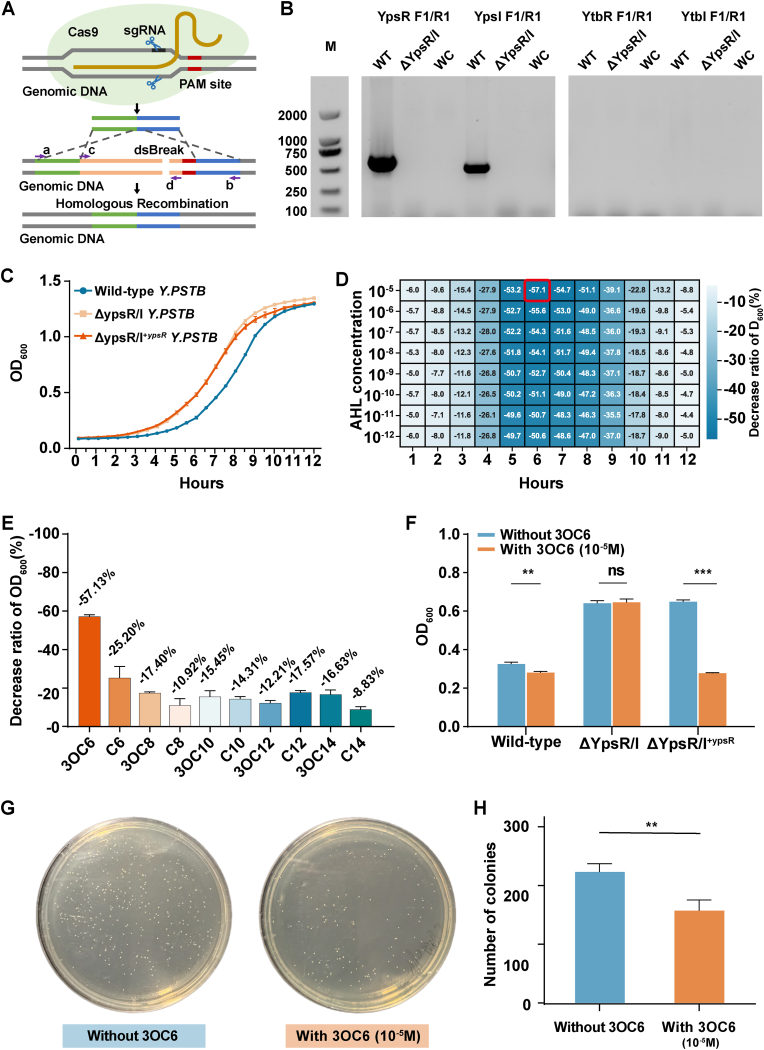
**Interaction and signal transduction ability of YpsR/YpsI with AHL small molecules.** (A) Schematic of the YpsR/YpsI gene knockout; (B) Verification of the *ypsR/ypsI* gene and *ytbR/ytbI* gene knockout with primers; WT refers to the *Y. pseudotuberculosis* wild-type strain amplified product, and WC refers to the water control amplified product; (C) Wild-type, ΔYpsR/YpsI and ΔYpsR/YpsI ^*+ ypsR*^ growth curves; (D) Growth of *ypsR* rescued bacteria (ΔYpsR/YpsI ^*+ ypsR*^) under different concentrations of 3OC6; (E) Decrease ratio of *ypsR* rescued bacteria (ΔYpsR/YpsI ^*+ ypsR*^) cultured with 10 AHL small molecules for 6 h; (F) Inhibitory effects of 5–10 M 3OC6 on the three bacterial strains for 6 h; plate counting experiment of (G, H) *ypsR* complemented bacteria (ΔYpsR/YpsI ^*+ ypsR*^) at 10^−5^ M 3OC6 (n = 3, *∗ ∗P ≤ 0.001, ∗ ∗ ∗P ≤ 0.01*).

In *Yersinia pseudotuberculosis*, QS involves two pairs of LuxR/LuxI orthologs (YpsR/YpsI and YtbR/YtbI) and multiple N-acylhomoserine lactones (AHLs). To investigate the QS system's role in the growth of *Y. pseudotuberculosis*, we used the CRISPR/Cas9 system to knock out YpsR/YpsI, while the *ytbR/ytbI* genes were not present in the strain (Fig. 3A and B). We constructed wild-type strains, YpsR/YpsI knockout (ΔYpsR/YpsI), and YpsR-rescued (ΔYpsR/YpsI + ^*ypsR*^) mutants. Growth curves revealed that the wild-type strain exhibited a significantly slower growth rate than that of the knockout strains, confirming that the YpsR/YpsI system influences the growth process of *Y. pstb* (Fig. 3C).

The YpsR-rescued strain displayed a growth phenotype similar to that of the double-knockout strain. Considering that the absence of YpsI affects the synthesis and accumulation, thus impacting YpsR's regulatory ability, we hypothesized that exogenous AHL supplementation could alter the growth pattern of the YpsR-rescued strain. Upon adding various AHLs to the medium, we observed that the YpsR-rescued strain's growth was generally slower than that of the YpsR-rescued strains without AHLs, particularly between 5 and 8 h (Fig. 3D, Fig. S4). Notably, 3OC6 exerted a significantly stronger growth retardation effect than other AHLs. Compared to the ΔYpsR/YpsI + ^*ypsR*^ strain cultured alone, the biomass accumulated at 6 h was 57 % lower when the strain was cultured with 10^−5^ M 3OC6-HSL; in contrast, other AHLs reduced biomass by at most 25 % (C6, Fig. 3E). This AHL-dependent growth retardation shifted the YpsR-rescued strain's growth pattern to resemble that of the wild-type strain than the double-knockout strain. The double-knockout strain's growth pattern was unaffected by exogenous AHL addition; indicating that AHL-mediated growth retardation occurs through interaction with YpsR (Fig. 3F). Colony counting and spreading experiments further confirmed the AHL molecules' growth inhibitory effect (Fig. 3G & H). Overall, these growth phenotype experiments demonstrated YpsR's interaction with AHL molecules in vivo, with growth retardation reflecting the interaction strength between the different AHL molecules and YpsR, consistent with previous in vitro biochemical assays.

### 2.4 Construction of an AHL-induced QS circuit in YpsR

**Fig. 4:**
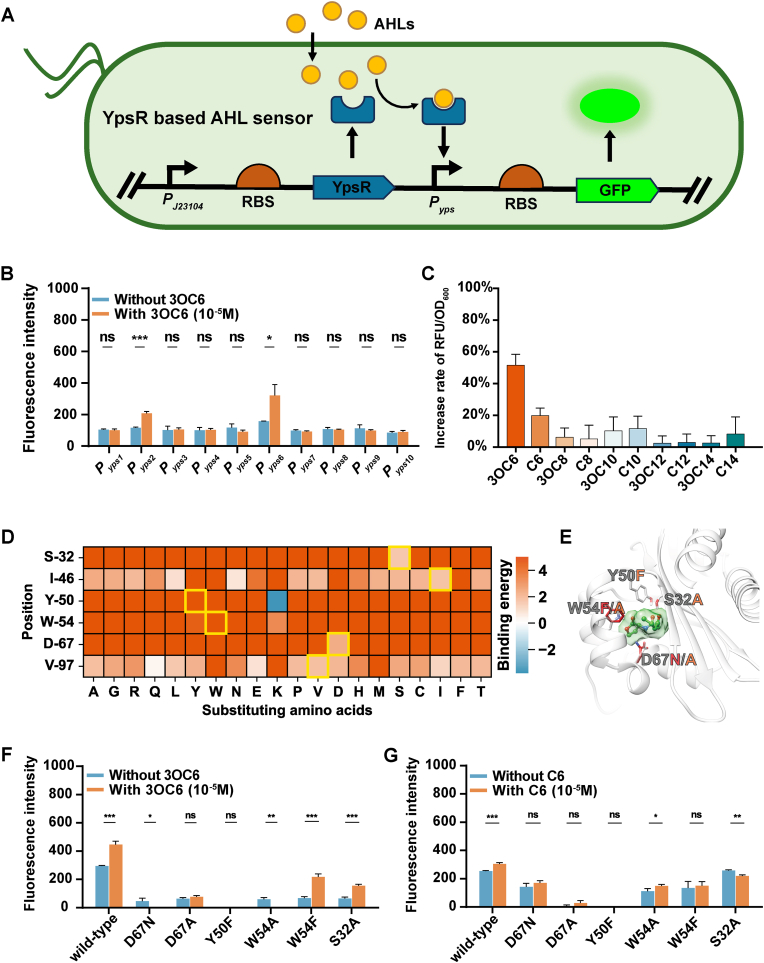
**Genetic circuit design and mutation site simulation based on YpsR.** (A) Gene circuits were designed and synthesized using components in the standard biological parts of the library; here, YpsR was used as the sensor protein, and GFP was used as the reporter protein. (B) Activation effects of 10^−5^ M 3OC6 on 10 whole-cell biosensors. (C) Activation effects of 10 AHLs on the whole-cell biosensor based on *Pyps6*. (D) Virtual mutation scanning heatmap of the YpsR protein complex and 3OC6. The heatmap shows the calculated binding energy changes of 20 single mutations within the binding site. The rotation and binding energy after mutation were calculated using PLOP; the color bar represents kcal/mol. The wild-type state of the site is represented by a yellow box. (E) The key residues of ligand binding for mutational analysis. The ligand is colored green with the surface rendered. (F, G) Response effects of the YpsR mutant with 3OC6 and C6 (n = 3, *∗ ∗P ≤ 0.001, ∗ ∗ ∗P ≤ 0.01*).

Building on our prior evidence of YpsR's ability to bind AHL molecules, we sought to verify the impact of YpsR-AHL interactions on downstream gene regulation by heterologously constructing a functional genetic circuit in a chassis strain other than *Y. pstb*. We selected the commonly used *E. coli* DH5α as the chassis, as it lacks QS systems that could interfere with YpsR testing, ensuring orthogonality between the genetic circuit and the host chassis. In this genetic circuit, the constitutive promoter J23104 drives *ypsR* expression, while the *gfp* gene is regulated by a YpsR-resposive promoter regulated. GFP fluorescence level indicates the activity of this regulatory promoter. Based on the LuxR transcription factor family's mechanism of action, binding with AHL molecules enhances the transcription factor's affinity promoter regions, recruiting RNA polymerase to promote downstream genes transcription—effectively creating an AHL sensor device based on YpsR (Fig. 4A). Drawing from the literature, we selected ten potential YpsR-regulated promoters, *PypsX* (*X* from 1 to 10), to construct ten AHL sensors (Table S1).

We first tested the sensor's capabilities with 3OC6-HSL, previously validated as having the strongest YpsR binding activity. Culturing conditions with and without 3OC6-HSL were employed to measure relative fluorescence intensities. the sensor utilizing the Pyps6 promoter exhibited the highest fluorescence intensity in the presence of AHL molecules and showed a significant difference compared to conditions without AHL. Additionally, the *Pyps2*-based sensor demonstrated significant AHL-sensing capability, although with lower absolute fluorescence intensity than *Pyps6* (Fig. 4B). We further used the *Pyps6*-based sensor to detect various AHLs. Only 3OC6-HSL significantly induced GFP expression, increasing fluorescence by approximately 50 %, whereas other AHLs increased fluorescence by less than 20 %, with an average increase of less than 10 % (C6, Fig. 4C). Thus, by leveraging YpsR-AHL interactions to regulate downstream genes, we successfully constructed an AHL sensor genetic circuit and verified its specific response to different types of AHL molecules.

### 2.5 Hot spot residues identified by mutational analysis

To elucidate the interaction profile between YpsR and 3OC6, we employed molecular mechanics with generalized Born and surface area solvation (MM-GB/SA) methods to calculate the binding energy differences for the mutants of key binding site residues within the YpsR binding site for 3OC6. Following a qualitative assessment of the protein‒ligand interactions in the AHL binding site, we performed a virtual mutational scan targeting residues located within 4 Å of the ligand. The resulting heatmap in Fig. 4D illustrated the differences of binding energy for 20 single-residue mutations within the binding site. Notably, mutations of the polar residues—Ser32, Tyr50, Trp54, and Asp67, all integral to hydrogen bonding—leading to a significant increase in binding energy, suggesting a detrimental impact on binding affinity. Conversely, mutations in apolar residues exhibited less pronounced changes in binding energy.

Among these, the Y50K mutation was observed to decrease the predicted binding energy. However, introducing a net charge for current binding site can cause complex effects, making predictions less reliable. Numerous studies have shown that altering charged residues in binding sites can significantly impact conformation [31]*,* dynamic properties [32], and substrate binding [33], thereby influencing overall function [[34], [35], [36]]*.* Given the relatively hydrophobic nature of the YpsR binding site, the introduction of charged residues in an apolar environment could generate strong energetic potentials. Additionally, charge−charge interactions, which can be significant over long-range distances up to 10 Å, might influence the conformation and stability of the binding site, particularly through Interactions with nearby Arg30 and the oppositely charged Asp67, crucial for ligand binding. Consequently, the S32A, Y50F, W54 F/A, and D67 N/A mutations were selected for further experimental investigation (Fig. S5). The mutated residues are highlighted in Fig. 4E. As shown in Fig. 4F and G, only W54F and S32A mutants exhibited noticeable activation effects following the addition of AHL molecules (3OC6 and C6) at a concentrations of 10^−5^ M. However, the fluorescence intensities for YpsR and 3OC6 were lower compared to that of wild-type YpsR, indicating that natural YpsR possesses strongest binding affinity for 3OC6. The significant reduction in GFP production observed in the S32A-3OC6 complex, in contrast to the wild type and the minimal changes seen with other AHLs, underscores the crucial role of the hydrogen bond formed by Ser32 in stabilizing 3OC6 binding. These results suggest a potential loss of binding capacity for larger Cx ligands and 3OCx, corroborating the results from the molecular interaction analyses. The variation in GFP production in the W54F mutant, coupled with its distinct behavior from W54A, emphasizes the importance of the hydrogen bond donor capacity and the spatial occupation of the aromatic ring in stabilizing AHLs. The absence of activation in the D67A and Y50F complexes with all AHLs highlights the critical role of these conserved polar hydrogen bond donors and acceptors in ligand binding.

### 2.6 Design and application of the whole-cell biosensor for *Y. pstb* detection

**Fig. 5:**
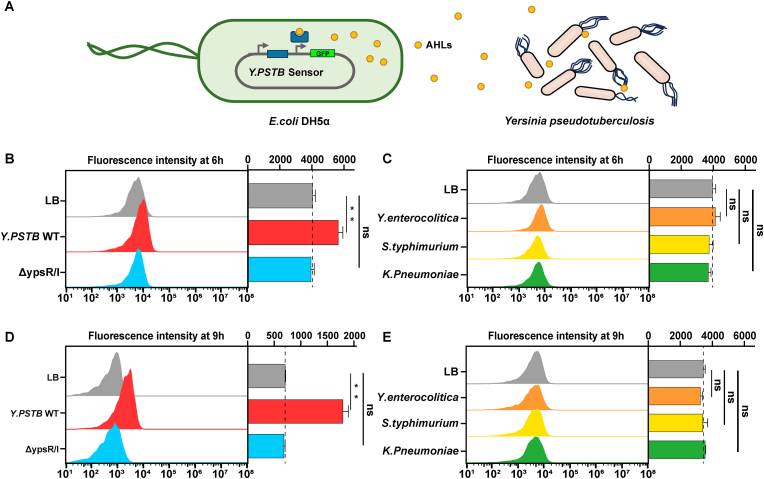
(A) Schematic diagram of the YpsR-AHL-based biosensor and *Y. pstb* coculture. (B) Relative fluorescence intensities of the biosensors cocultured with *Y. pstb* and ΔyspR/I for 6 h. (C) Relative fluorescence intensities of the biosensors cocultured with *Yersinia enterocolitica*, Salmonella typhimurium and *Klebsiella pneumoniae* for 6 h. (D) Relative fluorescence intensities of the biosensors cocultured with *Y. pstb* and ΔYpsR/YpsI for 9 h. (E) Relative fluorescence intensities of the biosensors cocultured with *Yersinia enterocolitica*, Salmonella typhimurium and *Klebsiella pneumoniae* for 9 h (n = 3, *∗ ∗P ≤ 0.001, ∗ ∗ ∗P ≤ 0.01*).

Finally, we investigated the ability of the AHL sensor to directly detect the AHL molecules produced by sample strains. These sensors can be employed as whole-cell biosensors for the detection of molecular markers specific to bacterial pathogens (Fig. 5A). The DH5α AHL sensor was co-cultured with wild-type *Y. pstb* or the ΔYpsR/YpsI double-knockout strain at a sensor-to-sample ratio of 1000:1, followed by flow cytometry analysis after 6 or 9 h. The sensors co-cultured with wild-type *Y. pstb* exhibited significant fluorescence (Fig. 5B and D). This fluorescence was markedly higher than that observed when co-cultured with the ΔYpsR/YpsI double-knockout strain or when cultured alone, with no significant difference detected between the latter two conditions. The enhanced fluorescence levels observed at 9 h compared to 6 h can be attributed to the synthesis and accumulation of AHL molecules by wild-type *Y. pstb* during culture. We further utilized the whole-cell sensor to measure fluorescence levels of other pathogens under the same conditions, including *Yersinia enterocolitica*, *Salmonella typhimurium* and *Klebsiella pneumoniae*, none of which are reported to synthesize 3OC6-HSL. When co-cultured with these strains, the whole-cell sensor exhibited fluorescence level consistent with those observed when the sensor was cultured alone, without the significant increase seen when co-cultured with wild-type *Y. pstb* (Fig. 5C and E). In summary, YpsR-based whole-cell biosensors can effectively and specifically detect AHL biomarkers from target strains, thereby distinguishing these strains from non-target phenotypes within the same species or from other species. This method holds potential for development into a point-of-care testing (POCT) device for the sensitive and specific detection of pathogens.

## 3 Discussion

QS systems are widely recognized as critical in orchestrating and coordinating bacterial collective behaviors, particularly in regulating the expression of virulence factors. These systems play key roles in processes such as biofilm formation and the enhancement of bacterial antibiotic resistance. Despite their importance, the molecular mechanisms underlying QS signal transduction pathways in pathogenic bacteria remain poorly understood, and comprehensive, systematic studies are still lacking. Furthermore, due to the common structural frameworks and biosynthetic pathways shared by AHL-based QS molecules, elucidating the mechanisms of their molecular recognition specificity has been a significant challenge.

This study focuses on the YpsI/YpsR QS signaling pathway in *Y. pseudotuberculosis* and, for the first time, systematically elucidates the molecular basis of the interaction between QS signaling molecules and the YpsR transcriptional regulator. Advances in deep learning-based protein structure prediction algorithms, such as AlphaFold, have significantly advanced propelled the fields of structural and computational biology. In the absence of experimentally determined structures, the high-confidence predictions provided by AlphaFold have offered a solid foundation for further research. In our study, the predicted structure of the cytoplasmic membrane-associated regulatory domain of YpsR, with an average pLDDT of 84.7, demonstrated reliable structural accuracy. The strong correlation between the predicted binding affinities and those obtained from SPR analysis, along with the observed ligand responses in YpsR mutant, clarifies the ligand binding mode and offers critical insights into the molecular mechanism underlying the pathogenicity of *Y. pstb*. Leveraging our deep understanding of the molecular recognition capabilities of YpsR, we developed an AHL sensor based on YpsR and applied it to whole-cell biosensing for *Y. pseudotuberculosis*. A critical aspect of biosensor design is maintaining the functional robustness of the sensor device, particularly its recognition specificity. The AHL sensor we developed was functional in a heterologous host, *E. coli*, while retaining specificity for 3OC6-HSL. This finding underscores the functional robustness of the YpsR regulatory protein and its ability to mediate AHL signal transduction. While the deployment of laboratory-developed biological devices in real-world applications often encounter challenges due to the complexity of real-world samples, our YpsR-based AHL sensor successfully identified the initial presence of *Y. pseudotuberculosis* in samples under extremely low inoculation conditions, distinguishing it from other pathogenic bacteria. This result suggests the preliminary feasibility of using QS signal molecules as biomarkers and highlights the potential application of this whole-cell sensor in point-of-care testing (POCT). This study lays essential groundwork for the prevention, control, and treatment of *Y. pseudotuberculosis*.

Despite our success in characterizing the binding affinities, the underlying reasons for the strength of interaction between YpsR and AHLs remain unclear. Consequently, we employed computational structure modeling, molecular docking and molecular dynamics simulations to predict the YpsR-AHL complex structures. Furthermore, research on other QS systems of *Y. pseudotuberculosis* remain scarce. The factors influencing the regulation of AHLs, such as 3OC6 during the YpsR-mediated tuberculosis process, are not yet fully understood. Thus, rational design of a single YpsR/YpsI QS system remains challenging. In the current study, the natural YpsR protein exhibited a stronger binding affinity to AHLs compared to YpsR single-point mutants, which may limit the construction of a highly sensitive and stable biosensor for *Yersinia*. Moving forward, we plan to screen for mutants with enhanced YpsR-AHLs binding capabilities through directed mutagenesis to expand the application range of the YpsR/YpsI QS system and to improve the potential for preventing *Yersinia* outbreaks. Additionally, while our computational results are promising, an experimentally resolved structure of YpsR-ligand complex would further validate our findings and provide critical molecular details relevant to its function.

The positive outcomes of this work underscore the importance of integrating modern artificial intelligence technologies and physics-based simulations. By leveraging predictive and post-processing models, we have laid the groundwork for discovering high-affinity ligands and advancing more sensitive and stable biosensors for *Yersinia*. Through the use of in silico molecular simulations, in vitro thermodynamic experiments, and in vivo growth rate assays, we have comprehensively characterized these interactions. Furthermore, our study is the first to reveal that YpsR exhibits broad and differential recognition abilities for various QS signal molecules, with relatively high specificity for 3OC6-AHL. This specific recognition capability was consistently demonstrated through molecular simulations, in vitro biochemical validation, and in vivo growth rate inhibition assays. Our research offers a systematic and replicable paradigm for studying signal transduction between signal molecules and regulatory factors.

## 4 Materials and methods

### 4.1 Chemicals and bacterial growth conditions

Ten AHLs were purchased from Sigma–Aldrich (St. Louis, MO, USA). Stock solutions were prepared by dissolving appropriate amounts of the compound in nuclease-free water and subsequently preserved at −40 °C. *Escherichia coli* strains were cultivated at 37 °C in Luria Bertani (LB) medium or on LB agar plates supplemented with 50 or 100 mg/mL ampicillin. In the gene circuit assay, a fresh 5 mL LB medium supplemented with ampicillin was inoculated with a single *E. coli* colony harboring the plasmid encoding the circuit (see section 2.4 of the main manuscript). The cells were incubated overnight at 37 °C with agitation at 220 rpm.

### 4.2 Gene verification of *Y. pseudotuberculosis* and ΔYpsR/YpsI

Four pairs of primers were designed and synthesized to differentiate wild-type and knockout bacteria (Table S2). The primers used were YtbR-F1 and YtbR-R1, YtbI-F1 and YtbI-R1, YpsR-F1 and YpsR-R1, and YpsI-F1 and YpsI-R1, with an amplification systems and various procedures employed. The target gene was identified through electrophoresis subsequent to colony PCR.

### 4.3 Preparation and transformation of *Y. pseudotuberculosis-* and ΔYpsR/YpsI-competent cells

A single colony from a plate was picked and inoculated into 5 mL of LB medium without antibiotics in a 50 mL shaking flask, followed by overnight incubation at 37 °C with agitation at 220 rpm. The subsequent morning, 1 mL of the overnight culture was transferred into 50 mL of LB medium and cultured at 37 °C with agitation at 220 rpm for 2 h until the OD_600_ reached approximately 0.6. The cells were then harvested by centrifugation at 5000 rpm, and the cell pellet underwent three washes with 10 mL of prechilled sterile water. After centrifugation at 4 °C and 3000×*g* for 2 min, the supernatant was decanted, and the cell pellet was resuspended in 1 mL of prechilled 10 % glycerol. The competent cells were aliquoted into 1.5 mL sterile EP tubes (100 μL per tube) and stored at −80 °C for future use. For transformation, 100 ng aliquots of pBAD-ypeR and pBAD-yspR plasmids were introduced to *Yersinia pseudotuberculosis* and *Y. pseudotuberculosis* ΔYpsR/YpsI competent cells, gently mixed, and transferred to the base of an electroporation cuvette using a pipette. The cuvette was promptly covered and placed on ice for 5 min. Subsequently, electroporation was carried out, followed by the addition of 1 mL of LB medium. After gentle mixing, the cells were left to recover at 37 °C with shaking at 220 rpm for 3 h. The transformed cells were then plated onto LB agar plates containing ampicillin and were incubated at 37 °C until colonies emerged. Individual colonies were selected, and PCR identification was conducted using the primers araBAD-F1 and GFP-R1 to screen for positive clones.

### 4.4 Model construction via AlphaFold prediction and molecular docking

The cytoplasmic membrane-linked regulatory domain of the YpsR structural model was forecasted employing AlphaFold [37]. Subsequently, ten distinct AHLs were singylarly docked into the ligand binding site using AutoDock Vina [38]. Among the various poses generated for each ligand, we chose the highest-ranked one based on the Vina score, a metric that demonstrates strong correlation with experimentally determined SPR binding affinity. All visualizations of the protein‒ligand complexes was conducted using Chimera [39].

### 4.5 Molecular dynamics simulation

All-atom molecular dynamics (MD) simulations were conducted using Gromacs 2023.2 [40] with the CHARMM36 force field for the protein and the CHARMM general force field for the AHL ligands [41,42]. Each YpsR-AHL complex system was prepared by solvation in a TIP3P water box with appropriate counterions to neutralize the charge [43]. The system underwent an initial minimization using the steepest descent algorithm for a maximum of 10,000 steps, followed by equilibration under an NVT ensemble for 100 ps. Subsequently, the production simulation was conducted in an NPT ensemble at 300 K and 1 atm for 100 ns on the equilibrated structures. Temperature and pressure were maintained using a Nosé–Hoover thermostat and Parrinello–Rahman barostat [44,45]. Root mean square deviations (RMSDs) between the initial conformation as the reference state and the trajectory conformations, as well as the root mean square fluctuations (RMSFs) of all protein *Cα* atoms, were computed using the MDTraj python library [46]. Conformational states for each AHL complex system were selected for further analysis of the ligand binding mode using LigPlot + [47].

### 4.6 Virtual mutational scanning

A virtual mutational scanning analysis was performed targeting the interaction residues in the ligand binding site of the YspR-3OC6 complex, based on MD simulation data. Each residue in the binding site was systematically mutated to all possible substitutional amino acids, with rotamers predicted using the Protein Local Optimization Program (PLOP) [48,49]. The resulting structures were optimized using the generalized Born implicit solvent model with the all-atom OPLS 2005 force field, and binding energies were calculated accordingly [50,51].

### 4.7 Protein expression and binding affinity measurement

The pMAT9S-based plasmids encoding YpsR–MBP fusions and their corresponding mutants were introduced into *E. coli* BL21 (DE3) competent cells. Cultures were cultivated in LB medium supplemented with ampicillin at 310 K until the optical density at 600 nm reached 0.8. Subsequently, isopropyl-β-d-thiogalactopyranoside was added to a final concentration of 0.4 mM, and the culture was continued at 289 K for 16 h. The cells were then harvested by centrifugation, and the bacterial pellets were resuspended in phosphate-buffered saline (140 mM NaCl, 10 mM Na_2_HPO_4_, 2.7 mM KCl, and 1.8 mM KH_2_PO_4_, pH 7.3) supplemented with 15 % glycerol. After high-pressure crushing at 277 K, the bacterial lysates were centrifuged at 18,000×*g* for 40 min at 277 K, and the pellet was discarded. The supernatant was purified by loading onto a amylose resin column (New England Biolabs, cat. no. E8021S) to isolate the MBP-tagged YpsR fusion proteins. Elusion was achieved using 20 mM maltose dissolved in resuspension buffer, followed by further purification through anion-exchange chromatography using a HiTrap Q column (GE Healthcare) and gel filtration chromatography using a Superdex-200 column (GE Healthcare) in resuspension buffer with a linear NaCl gradient (25–250 mM) in 20 mM Tris-HCl (pH 8.0) and 15 % glycerol.

We obtained the YpsR gene through gene synthesis, designed an expression vector, fine-tuned the boundaries, and optimized the expression in *Escherichia coli*. Subsequently, we purified the stable protein through experimental validation. Specifically, we chose N-terminal MBP-tagged and C-terminal His-tagged YpsR proteins spanning amino acids 1–179 (Fig. S3A). Furthermore, we conducted liquid chromatography‒mass spectrometry (LC–MS) intact mass analysis and peptide mass fingerprinting on the purified protein to validate the integrity and high purity of the YpsR 1–179 protein (Figs. S3B and S3C). The YpsR family of transcription factors is prone to aggregation in the absence of specific conditions, limiting direct biochemical characterization of the binding affinity between YpsR and 3OC6, aside from a rough dissociation constant estimate derived from a model fitted to luminescence data. To overcome this hurdle, we employed the rigid maltose-binding protein (MBP) technique to produce stable Apo-YpsR. Specifically, a rigid helix was appended at the C-terminus of MBP, restricting the flexibility of the attached recombinant proteins and enhancing their solubility.

### 4.8 Genetic circuit construction

Plasmid constructs were assembled in accordance with the BioBrick RCF standard employing a diverse assay of molecular cloning techniques. Biological components were acquired from the Registry of Standard Biological parts or synthesized chemically. The constitutive promoter PCon (BBa_J23104) coupled with a ribosome binding site (RBS; BBa_B0034) was integrated upstream of the synthetic *Y. pseudotuberculosis* YpsR gene to form the YpsR sender module. Downstream of the *YpsR*-HSL-regulated promoter (BBa_C0062) *PypsR*, the RBS (BBa_B0034)–GFP (BBa_E0040)–terminator (BBa_B0015) ensemble was inserted as the *YpsR* receiver module. The modules were assembled into the vector of pSB1A3.

### 4.9 Design and application of a whole-cell biosensor

Overnight *E. coli* cultures (refer to Supplementary Methods) were diluted 1000-fold into fresh LB medium containing 100 μg/mL ampicillin and dispensed into 1.5-mL centrifuge tubes containing varyinngn concentrations of 3OC6 (0, 10^−5^, 10^−6^, 10^−7^, 10^−8^, 10^−9^, 10^−10^, 10^−11^, or 10^−12^ M). The samples underwent an 8 h incubation at 37 °C before being transferred to a 96-well microplate for fluorescence measurement using a Synergy H1 hybrid Reader (excitation, 488 nm; emission, 518 nm). Optical measurements at 600 nm were taken to determine cell densities. Mean fluorescence values were calculated based on a minimum of three biological replicates. Furthermore, a bacterial dilution of 1:1000 and 3OC6 (10^−5^ M) were added to Corning Costar transparent bottom 96-well plates. The samples were subjected to continuous incubation in the Synergy H1 hybrid Reader continuously for 8 h (807 rpm, orbital shaking), with fluorescence intensity and OD_600_ readings recorded at 30 min intervals. The GFP production levels of the whole-cell biosensor in the presence of culture supernatants from various bacterial pathogens were evaluated. Overnight *E. coli* DH5α cultures were diluted 1:100 and grow to logarithmic growth phase under the same conditions. Biosensor cells were harvested by centrifugation at 3000×*g* for 4 min, resuspended in an appropriate volume of LB medium to achieve an OD_600_ of 1. For the detection of native 3OC6, *Y. pseudotuberculosis*, ΔYpsR/I, *Yersinia enterocolitica*, *Salmonella typhimurium*, and *Klebsiella pneumoniae* strains were collected at 8000×*g* for 5 min and co-cultured with the biosensor cells at a volume ratio of 1000:1 for 6 and 9 h at 37 °C. GFP fluorescence intensity and OD_600_ measurements were taken at 30-min intervals using a microplate reader.

## CRediT authorship contribution statement

**Boyu Luo:** Writing – original draft, Visualization, Validation, Project administration, Methodology, Investigation, Formal analysis, Data curation. **Shanshan Wu:** Validation, Software, Project administration, Methodology, Investigation, Formal analysis, Data curation. **Wei Liu:** Methodology, Investigation. **Dongdong Zhang:** Project administration, Methodology, Investigation. **Ruicun Liu:** Visualization, Software, Methodology, Investigation, Formal analysis, Data curation. **Tuoyu Liu:** Software, Methodology, Investigation, Data curation. **Zhi Sun:** Supervision, Project administration, Methodology, Investigation, Funding acquisition, Xinyue Fan, Methodology, Investigation. **Ziqun Wei:** Validation. **Mingyu Liu:** Validation. **Zhiyuan Shi:** Conceptualization, Funding acquisition, Project administration, Supervision, Writing – original draft, Writing – review & editing. **Niu Huang:** Conceptualization, Project administration, Supervision, Writing – original draft, Writing – review & editing. **Yue Teng:** Writing – review & editing, Writing – original draft, Visualization, Validation, Supervision, Resources, Project administration, Funding acquisition, Data curation, Conceptualization.

## Declaration of competing interest

The authors declare that they have no known competing financial interests or personal relationships that could have appeared to influence the work reported in this paper.

☒The authors declare the following financial interests (e.g., any funding for the research project)/personal relationships (e.g., the author is an employee of a profitable company) which may be considered as potential competing interests:

The authors declare that they have no conflict of interest. The funders were not involved in the current analyses or in the preparation of this manuscript. The corresponding author has full access to all data and the final responsibility for the decision to submit for publication.
