# Supplementary figures and images for: Mechanistic insights into the orthogonal functionality of an AHL-mediated quorum-sensing circuit in Yersinia pseudotuberculosis
Source: Synth Syst Biotechnol. 2024 Oct 14;10(1):174–84. doi: 10.1016/j.synbio.2024.10.002 (PMC11564790; doi:10.1016/j.synbio.2024.10.002)

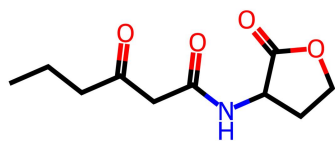

30C6

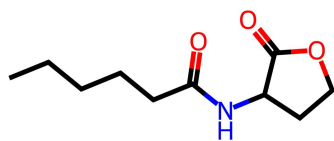

C6

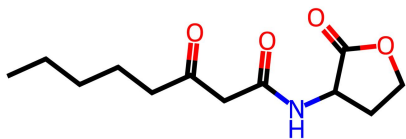

30C8

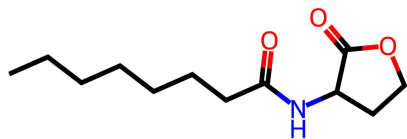

C8

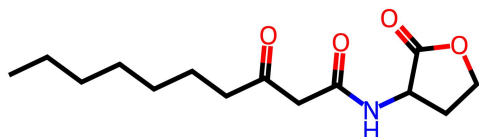

30C10

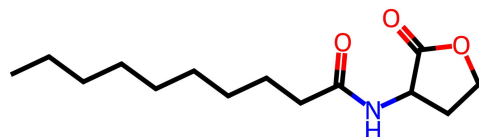

C10

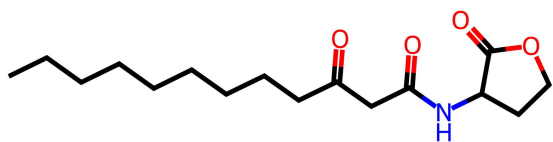

30C12

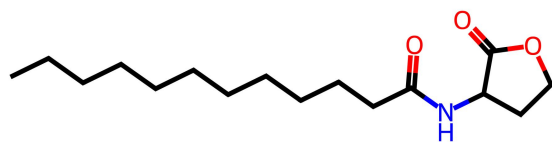

C12

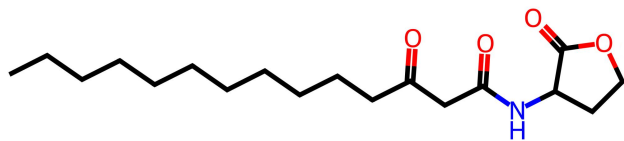

30C14

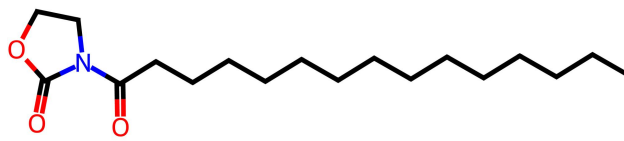

C14

Supplement: Multimedia component 1 [file mmc1.pdf]

**A**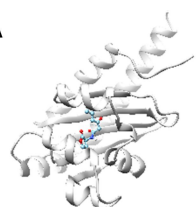**30C6**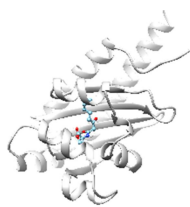**30C8**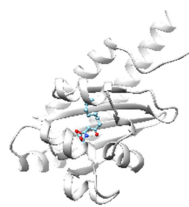**30C10**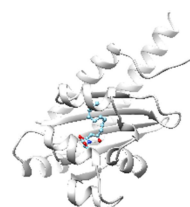**30C12**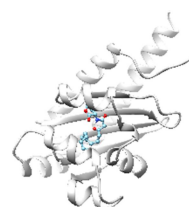**30C14**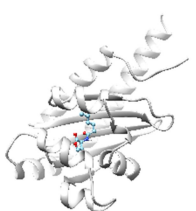**C6**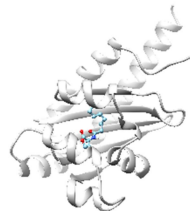**C8**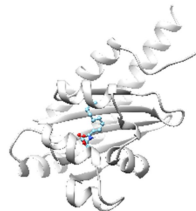**C10**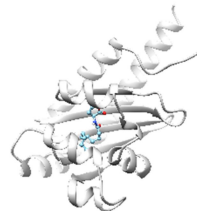**C12**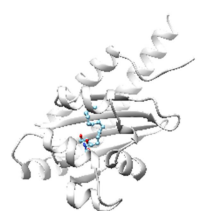**C14****B**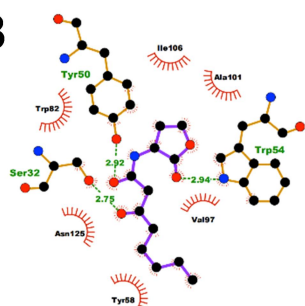**30C8**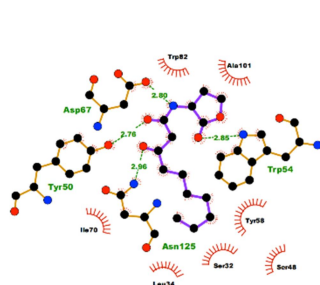**30C10**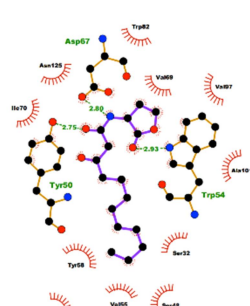**30C12**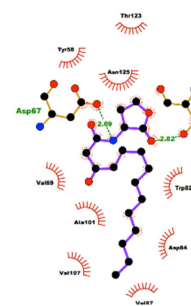**30C14**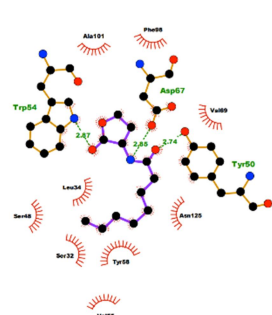**C8**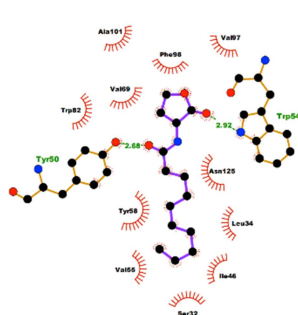**C10**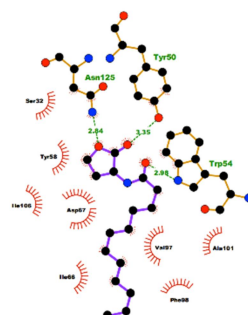**C12**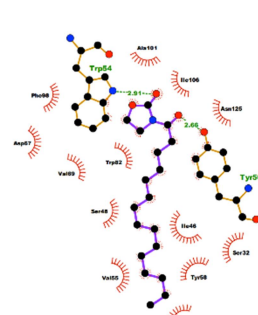**C14**

Supplement: Multimedia component 2 [file mmc2.pdf]

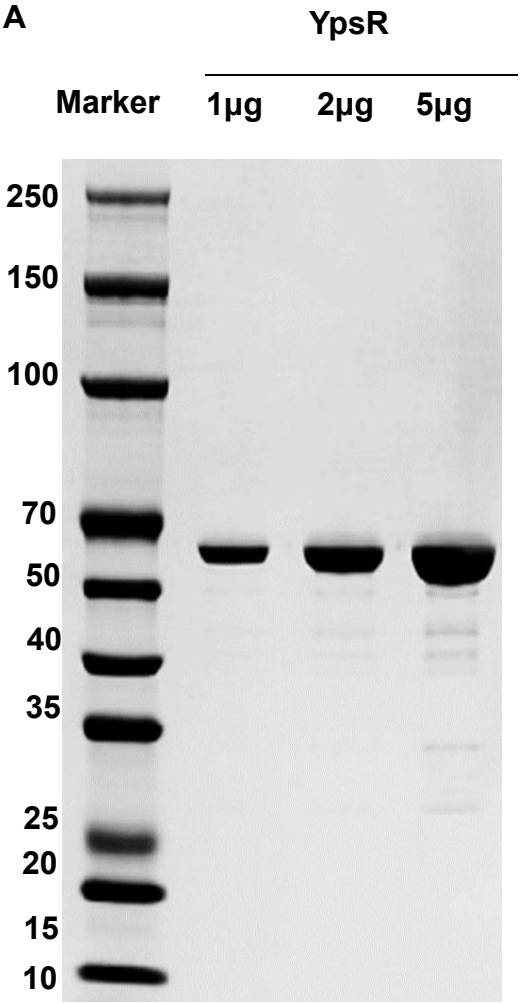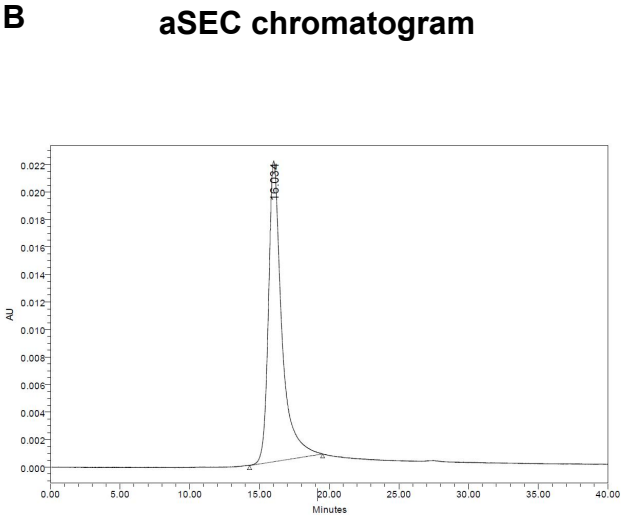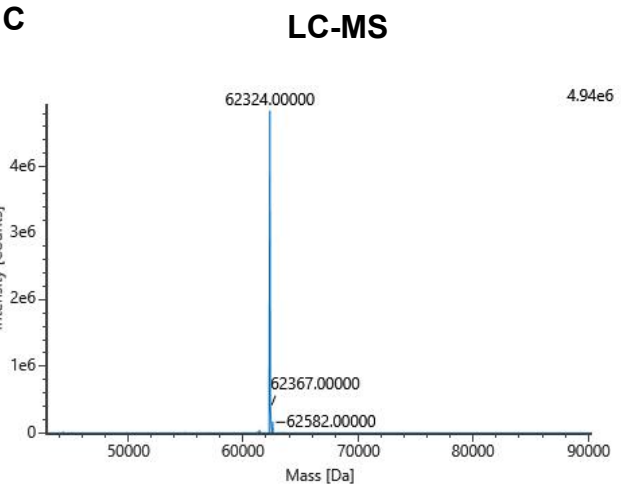

Supplement: Multimedia component 3 [file mmc3.pdf]

## 30C6

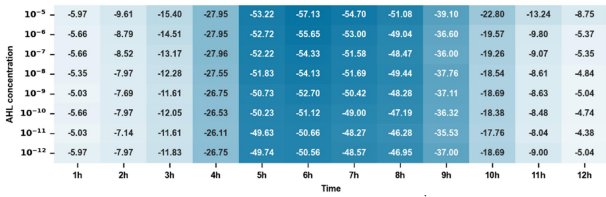

## C6

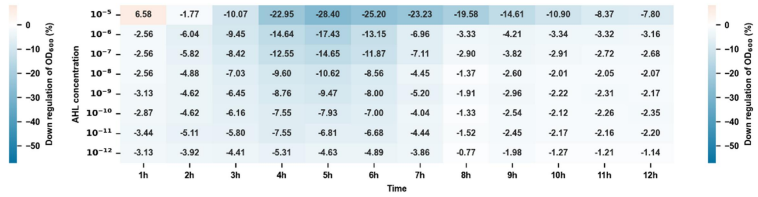

## 30C8

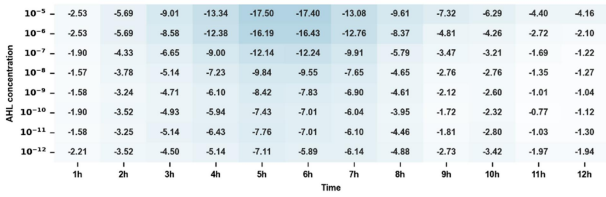

## C8

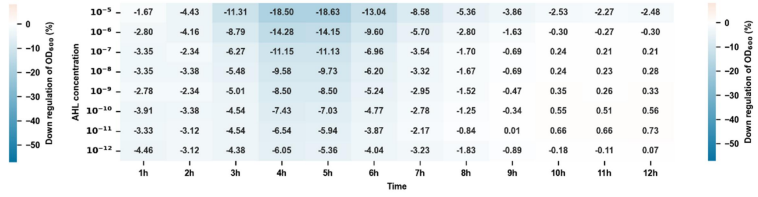

## 30C10

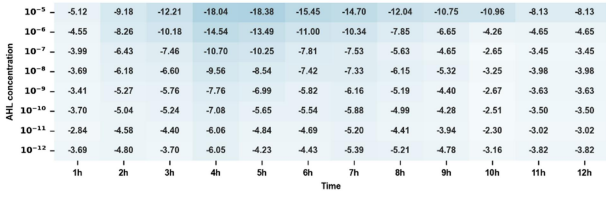

## C10

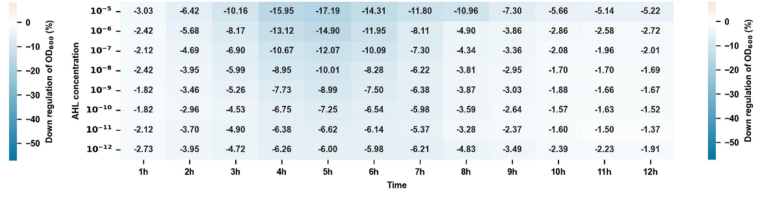

## 30C12

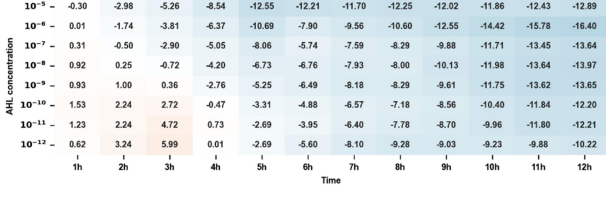

## C12

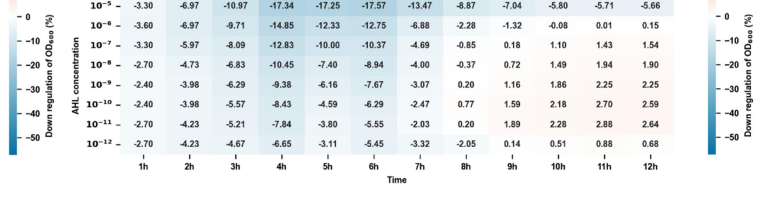

## 30C14

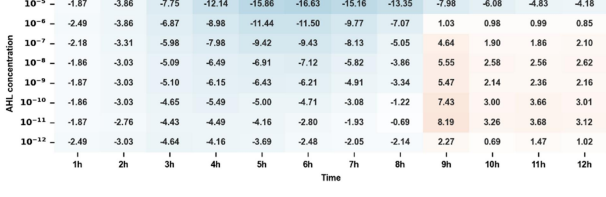

## C14

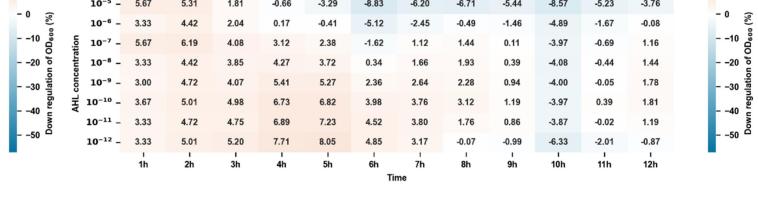

Supplement: Multimedia component 4 [file mmc4.pdf]

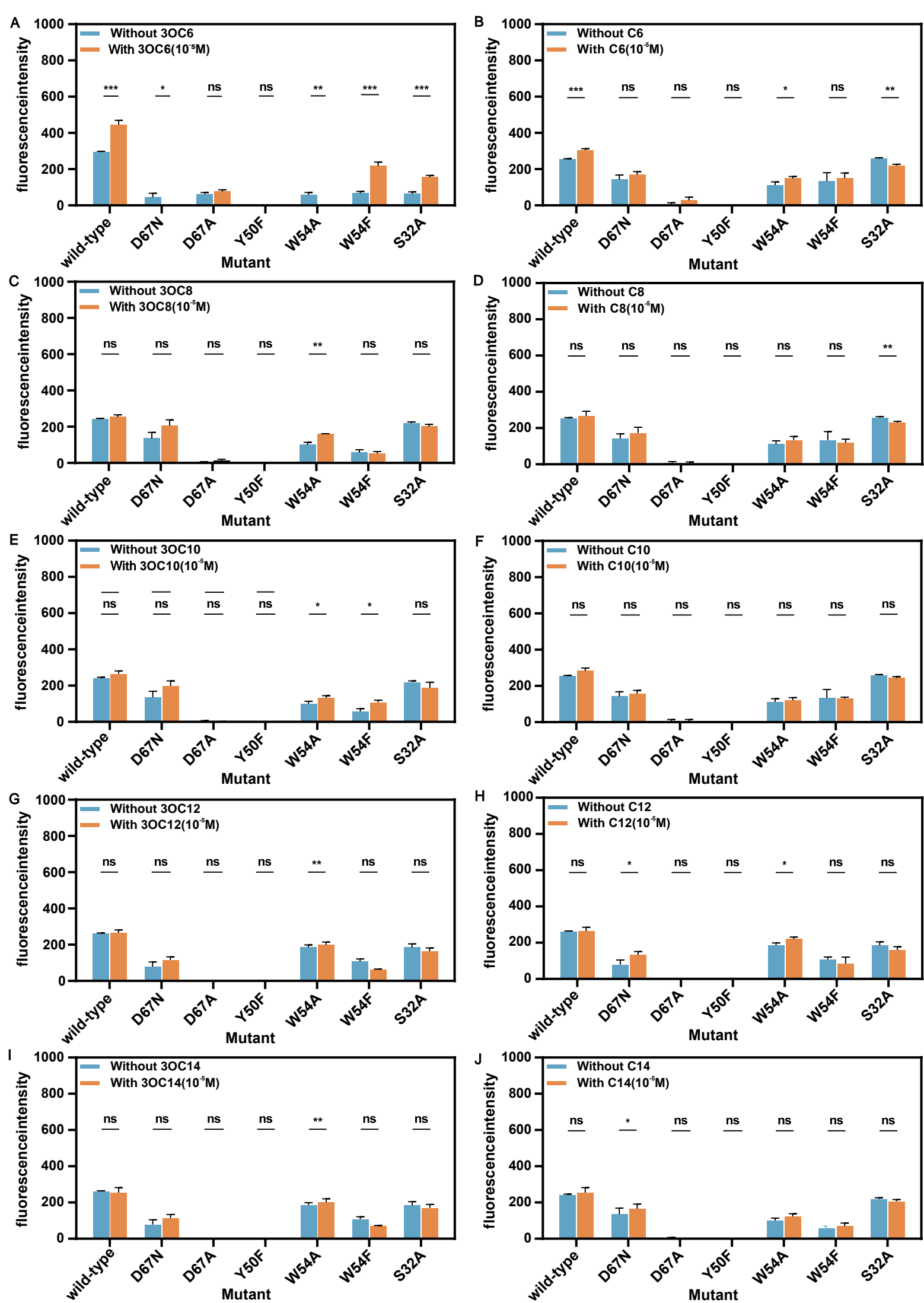

Supplement: Multimedia component 5 [file mmc5.pdf]
