# Supplementary material for: Mechanistic insights into the orthogonal functionality of an AHL-mediated quorum-sensing circuit in Yersinia pseudotuberculosis
Source: Synth Syst Biotechnol. 2024 Oct 14;10(1):174–84. doi: 10.1016/j.synbio.2024.10.002 (PMC11564790; doi:10.1016/j.synbio.2024.10.002)
Supplement: Multimedia component 6 [file mmc6.docx]

**Table S1.** The sequences of promoters

| **Promoters** | **Sequence** | | | | | | | |  |  |  |
| --- | --- | --- | --- | --- | --- | --- | --- | --- | --- | --- | --- |
| *Pyps1* | CCAATATCACCTTATAGTCAACACAGATGGAAAAATTTAGCGTGCCACTTCACATAGATTAAATTATGCGGCATAAGTTATGGTAAAGTTAATGTAGATTCTGAGGTTGGGTAGACGTACTTGTTTACCCGTTAACGGACTCCCAGAACTATTAGCAATCCAATTATTCCCTAATGTGACAACTTATTTTGTGATGATACAATACAATTCAATTTAACATATGCACACTGGCAGTATTAAATATCAGGAAAGATA | | | | | | | |  |  |  |
| *Pyps2* | AGGGGAGCTAGACATTATATTAAATGCTACAGAAGTTCTACTTGCAGAAACAGGTGCACTTCTGTAGCATTTTCCTTTATTATTTTTGGTTTTTCCCTGGTATTGACTAGACTTTTGTTGTCACCTGACTCAAGTAGCTAGGTTTTTTCATCAACTTACTCTATCCATTATACGGGAACGGGTATGGGGCGGTAGTTGTACTAAGGTGCAATTTCCTATTGCACCTTAGTACAGTAAATGGAGAAAGGGA | | | | | | | |  |  |  |
| *Pyps3* | TAATTACCTTTTCTGCTCTGTCCAAAGTCGTTGGCGCTGAGGCAGTGAAGCAAATGAGCGAATTTTGTAAAGTGGCTCATGTCAGTAACTCGGGTGAGACGGTGAGAAAAGGTGGTTACCCCCTGTAACGTAAAGTAATTAGGGTCTGTAAGGTCTAATTAACAATATAACGGGAAGGATCCCTGGTTTTGAGTGATAAATTTTGTCATTCAATTTATGTTTTAAAACATAATCAGTTGGGATGTGTAAG | | | | | | | |  |  |  |
| *Pyps4* | TTAACCAGATTGTCCAGGCAGTTAGTATGACAATGGGGGATTTTATCCCCCATTTCTTTTTCCTCGCATTGTTATCGTTGAATGTAAGACATTCGTCCCCATATACTCAAATACCTATTTAGTGAAACTCGTGATGACTCGTGTTTCACGAGATTTCCAGTAATCTGGCGGAAGCTAAACTAAGAGAGAGCTCT | | | | | | | |  |  |  |
| *Pyps5* | AGTAGGGATAAGAGTTGGTCGTTAGATAAAAGGTACCTGTGGGGCTTGAAAACACGTATTGATCCCCCAATTAAGCTAACAGACGTTTATATAAGATGTGATGAAACACTGGCTGATTATCATTGTCGAATGAGTCTGGGAGGCCAGGCGTTTTGTATGCGACCTTGTTGCATGAACATAGTCATCATTACTTATCTCAAGTAGAATCGGTTATGAATGGCGCCAAAGTAAATTCTATTAGGAGACGGTA | | | | | | | |  |  |  |
| *Pyps6* | CGACTTATTTCTGCGAGCCGATATACTATAGTGTATGTAAATAGAACAAATTATGGGATCGAGTAGATGCAATCGCGTGTTGTTCGAATACTCCTTGGTATATCGGCTATCCGTTGAACGTTAACGTGATGTCCAGAAATAGCGAGAATAGTCTCCCTGCCACCATAGTTTCGGGTGTGGCAGATACTAAAGAAGAGGTTTACTG | | | | | | | |  |  |  |
| *Pyps7* | GGGATAGTTCCACCAATATTAATTTGTATATCGAGAAGAAAAACCACC | | | | | | | |  |  |  |
| *Pyps8* | TTCATTTGTGATGAGTTTTAAAATAAAATACCCGGATGGAAGCCAGCTTTCCGAGTTAGTGGAAGAATATTTAGACGACACATATACTCTGTTTAGTAGTTATGGAATCAATGATCCTGAATTGCGTCGCTGGCAGAAAACCAAAGAGCATTTATTCAGACTTTTCTCAGGGGAATACGTATGTACCCTGATGAAAACTTAAGGGATAGTTCCACCAATATTAATTTGTATATCGAGAAGAAAAACCACC | | | | | | | |  |  |  |
| *Pyps9* | GAAAAGCCCCCTGGTGTTGGCTAAAATATATTCACAGCAGACTAACTTTTTCAGCAACAATAAGCTAAAATATTAGTTCTATTATATTAAGCACTTATTCTTTAAAGCAGAAATATTTTAGTTAACATAGGTCTATAATAGTAGCTCTATTTAAGTCTAGTTCTTAGTACTATCCTTTAGTCTAGGTTTCTTTTTTCAAGAATGTCAATAATTTGAATGTGATGCAGTGATAAAGTAAATTTGGTCTGCT | | | | | | | |  |  |  |
| *pyps10* | TTTATACCCTGAATAATTCGAATTGCAGGAAGGCGGCAAGCGAGAGAGCCCCGATGAGCTTACTGTAGTAAGTCATTCGGATGAATATAGGCCACGAGAATCATATAAATAGAAAGTTAAAAAGAGGCGATAACTCCGCATCATGCTTATCGCTATATTCATCTCATCACATAAAACACCTTCAACGTTTGTACTAACACTCTAAAATCATGTATGATATTGGTTTAATTAAATAAATTTGGTTTTTATT | | | | | | | |  |  |  |
|  |  |  |  |  |  |  |  |  | |  |  |
