# Supplementary material for: Mechanistic insights into the orthogonal functionality of an AHL-mediated quorum-sensing circuit in Yersinia pseudotuberculosis
Source: Synth Syst Biotechnol. 2024 Oct 14;10(1):174–84. doi: 10.1016/j.synbio.2024.10.002 (PMC11564790; doi:10.1016/j.synbio.2024.10.002)
Supplement: Multimedia component 7 [file mmc7.docx]

**Table S2.** Primers and sequences for colony identification.

| **gene** | **Prime** | **sequences** |
| --- | --- | --- |
| *ytbR* | F1 | CATTGTCACTAGCTACCCAAATGAATGGGC |
|  | R1 | CCTATGTGATATTTTACTGTTCTGGGGGTG |
| *ytbI* | F1 | CATTGTCACTAGCTACCCAAATGAATGGGC |
|  | R1 | CTTAATACTGAACATGGATCCTGCAAACGC |
| *ypsR* | F1 | GTACCGCTACATCCGGCGATCATATCGAAC |
|  | R1 | GCATGTTTGGCATTGAGAACACCTAAC |
| *ypsI* | F1 | CGTTTGGACTGGAAAGTAACCTGTATTG |
|  | R1 | CTGCTGTTGGCTATTACTGTCAATGCTC |
